# Dental Calculi of Siberian Natives, Russian Settlers, and Korean People of Joseon Dynasty Period in the 16th to 19th Century Eurasia Continent

**DOI:** 10.1155/2022/5765604

**Published:** 2022-05-09

**Authors:** Hyejin Lee, Jong Ha Hong, Larisa Tataurova, Sergey Slepchenko, Jieun Kim, Dong Hoon Shin

**Affiliations:** ^1^Department of Forensic Medicine, Seoul National University College of Medicine, Seoul, Republic of Korea; ^2^Ministry of National Defense Agency of KIA Recovery & Identification, Seoul, Republic of Korea; ^3^Institute of Korean Archaeology and Ancient History, Kyung Hee University, Seoul, Republic of Korea; ^4^Federal State Budgetary Institution of Science Institute of Archaeology and Ethnography of the Siberian Branch of the Russian Academy of Sciences, Omsk, Russia; ^5^Tyumen Scientific Center of the Siberian Branch of the Russian Academy of Sciences, Tyumen, Russia; ^6^Department of Anatomy and Cell Biology, Seoul National University College of Medicine, Seoul, Republic of Korea

## Abstract

**Objective:**

The prevalence of calculus is known to be variable by difference in diets or subsistence strategy between human populations. However, this situation has not been confirmed so far for hunter-gatherers and farming people in terms of history. In this study, we tried to reveal the association of diets or subsistence with calculus in different historical populations: Siberian natives, Joseon period Korean people, and Russian settlers in Siberia.

**Design:**

We examined the teeth of Siberian natives (hunter-gatherers), Russian (wheat farming) settlers, and Joseon (rice farming) people in sixteenth to nineteenth century. Age and sex were estimated using the methods of Buikstra and Ubelaker (1994). We examined specimens to detect signs of calculus formation in teeth. Calculus rates in each group were statistically compared, and the proportions of calculus by age or sex were also compared across each group. We used package R for statistical analysis. *Results and Discussion.* The prevalence of calculus deposition decreased in the order of Joseon people, Russian settlers, and Siberian natives. Our study proposes that the rate of calculi among farming people was evidently higher than that of hunter-gatherers in sixteenth to nineteenth century Eurasia. In all three groups, calculus prevalence became higher as age increases and was noteworthy in males.

**Conclusion:**

Current study demonstrated a significant difference of calculus formation between those groups with different diets or subsistence strategies. Higher prevalence of dental calculus was observed in agriculturalist Joseon Koreans and Russian settlers, but Siberian natives exhibited relatively lower frequency of dental calculus. The results of this study enable us to reconsider the meaning of association between subsistence strategy and calculus in different historical populations.

## 1. Introduction

Calculus is mineralized material on tooth surface that is covered by bacterial plaque. In general, it is the primary agent for various dental pathologies such as caries, periodontitis, and alveolar abscess [1–4]. The conditions that can affect calculus are very diverse [2, 5, 6]. Many factors like salivary flow, hydration, mineral and silicon content in food and water, and oral hygiene are known to affect the prevalence and extent of dental calculus in individuals. Clinical and experimental data also supported that urea and alkaline pH might induce calculus mineralization as well [7–9].

Besides them, the dietary pattern of specific population is one of the most serious causes of dental calculus forming [10]. Briefly, calculus forming might have been related to protein-rich food like fish or meat [7, 11, 12]. Others argued that carbohydrate-rich diets might promote calculus deposition [7, 13, 14]. Calculus is also facilitated by severe dental attrition that is known to be highly influenced by the type of food we eat [15–20]. Therefore, the association between specific populations' subsistence strategy and tooth pathology has been studied in different populations [21–28]. For example, the causal relationship between carbohydrates and caries has been discussed extensively. The more carbohydrate-rich foods they eat, the more cavities they might have [24, 29–36]. Likewise, the prevalence of calculus could be variable according to the difference in subsistence strategy and lifestyle of specific population [7]. However, the data of this situation, which could reveal information regarding the dental health and hygiene level of specific historical populations, has not been well established so far in terms of history.

The sixteenth to nineteenth century Eurasian continent is therefore an interesting topic for us. Since the wave of modernization was not yet dominated on the eastern part of continent until then, there were various population groups in this world that maintained their survival in very traditional ways. That is, the Siberian native people lived with hunting and gathering technique that had been handed down for generations. Many other people were also engaged in agriculture in Eurasian continent during the same period. Rice farming was a major production base for those Korean or Japanese people in East Asia. Russian settlers, mainly depending on wheat farming and lived relatively rudimentary, but under the clear influence of Russian Empire, also existed in Siberia. Although they existed in similar time and on the same Eurasian continent, they maintained completely different dietary and survival strategy. Since this means that the occurrence of calculus might have been quite different between them, we tried to reveal it with any scientific evidence.

## 2. Materials and Methods

**Figure 1:**
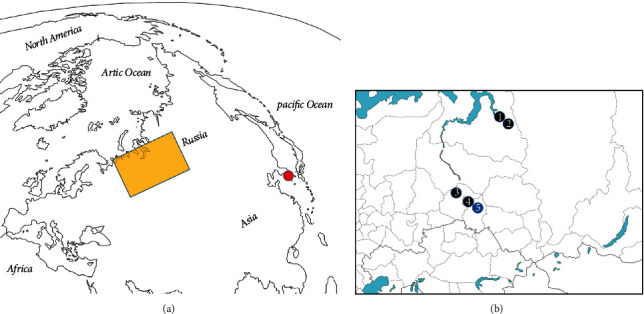
The geographic location of archaeological sites for each group studied in this research. (a) The red circle indicates South Korea. Yellow rectangle indicates the excavation sites of native and Russian settlers. (b) Magnified image of yellow rectangle part in (a). Black dots represent the sites of Siberian natives (1 and 2, Nenet; 3, Khanty; 4, Tatar). The blue dot indicates the excavation site (Izyuk, Omsk) for Russian settlers.

We examined the crania of West Siberian natives, Russian settlers, and Joseon period Korean people (Figure 1). All three groups were living in the sixteenth to nineteenth century. The archaeological information of Siberian natives, Russian settlers, and Joseon period mummies are summarized in Table 1 and Figure 1. As for the West Siberian natives, the crania have been curated by the Institute of the Problems of Northern Development Center (Tyumen, Russian Federation) [37]. The Siberian natives were hunter-gatherers. Their crania (*n* = 53; 30 females and 23 males) were originated from the Tatar (*n* = 34), Khanty (*n* = 7), and Nenet (*n* = 12) people (Table 1 and Figure 1). The total number of Siberian natives' teeth was 820. Among them, the Tatars, a Turkic-speaking people of West Siberia, were fishermen, hunters, and pastoralists [38]. The Khanty (fishermen and hunters) settled in north-taiga or forest-tundra zones at the Middle and Lower Ob River regions [39, 40]. The Nenets, as hunters, fishermen, and reindeer herders, lived at the Arctic and Near Arctic Circle [37, 41].

Meanwhile, the Russian settler's crania investigated in this study were consisted of 79 individuals (32 males and 47 females; total number of teeth = 1,304) (Table 1 and Figure 1). The Russian settlers' Izyuk village was built as early as 1648 CE around the Irtysh River [37]. A cemetery was found during archaeological excavation for the site. In previous reports, the settlers buried at the cemetery migrated from Central or Northern Russia as well as Eastern Europe [37, 42]. They were engaged in wheat cultivation. Their crania were curated in the Institute of the Problems of Northern Development Center (Tyumen, Russia) [37].

The Joseon dynasty people's crania were consisted of 90 individuals (48 males and 42 females; total number of teeth = 1,992) (Table 1 and Figure 1). They were engaged in rice cultivation. Joseon dynasty was one of the last countries to open its ports to Western countries. Therefore, our study on Joseon people's teeth can be a good example of dental pathology in a country where sociocultural changes by industrialization were delayed until relatively recent days [30]. The crania are curated in Seoul National University College of Medicine (Seoul, South Korea).

Age and sex were estimated by methods of Buikstra and Ubelaker [43]. Age estimation was based on pubic symphysis, auricular surface, ectocranial suture closure, and level of dental attrition. The age of immature remains was estimated using dental eruption and formation and epiphyseal closure of long bones. All individuals were grouped into 4 age categories for more detailed analysis: adolescents (15-19 years), young adults (20-34 years), middle adults (35-49 years), and old adults (over 50).

Sex was also estimated using dimorphic features in pelvis or skull, as these are reliable data for sex determination in osteoarchaeology. Skull's sexually dimorphic features include mastoid process, nuchal crest, inion protuberance, zygomatic root, supraorbital ridge, frontal shape, and gonial shape [44–47]. Dimorphic features of the pelvis also include ventral arc, preaurciular sulcus, greater sciatic notch, medial portion of the pubis, subpubic concavity, subpubic angle, and median ischiopubic ridge [47, 48]. The information of age and sex distributions for Siberian natives, Russian settlers, and Joseon people are summarized in Supplementary Tables 1 and 2, respectively.

Before our examination, every tooth was cleaned with soft brush to facilitate precise observation. All individuals were examined for any signs of calculus formation. Dental calculi were evaluated macroscopically under natural light with the aid of magnifying glass. Calculus was documented by the standards of Brothwell [45] and Buikstra and Ubelaker [43]. Two methods of analysis of dental calculi were used in this study, individual count method (per individual) and tooth count method (per teeth) [49]. Per individual method is useful in studying the population prevalence of a certain dental disease, and per teeth method permits larger sample sizes for statistical analysis and facilitates the comparison of disease frequencies [49]. The prevalence of calculi was also analyzed by sex and age to see any difference between them.

In this study, package R [50] was used for statistical analysis. We compared the proportions of age or sex across each group by Pearson's Chi-squared test [37]. The calculus rates in each group were compared by Pearson Chi-squared test. For comparison of prevalence in case that sample number was less than 10, Fisher's exact test was applied [37]. We used the package ggplot2 implemented in package R version 4.0.2 (R Foundation for Statistical Computing, Vienna, Austria) to draw charts for displaying prevalence of data in each group [51].

## 3. Results and Discussion

In statistical analysis to see the homogeneity in the age proportions, we confirmed that the age groups between Siberian natives, Russian settlers, and Joseon period Koreans were not differently distributed (Pearson's Chi-squared test, *P* value = 0.3225 and 0.217 for Siberian natives and Russian settlers and Russian settlers and Joseon people, respectively; Fisher's exact test, *P* value = 0.1507 for Siberian natives and Joseon people). Likewise, sex proportions showed no intergroup difference statistically between them (Pearson's Chi-squared test, *P* value = 0.1013, 0.3297, and 0.5157 for between Siberian natives and Russian settlers, Siberian natives and Joseon people, and Russian settlers and Joseon people, respectively).

In this study, the prevalence of calculus deposition *by individual* decreased in the order of Joseon people (76.7%), Russian settlers (60.8%), and Siberian natives (26.4%) (Table 2). Also, in the prevalence of calculus *by teeth*, the order was the same as seen in the prevalence by individual: Joseon people (32.5%), Russian settlers (29.2%), and Siberian natives (6.6%) (Table 3). In particular, the Siberian natives differed greatly from the other two groups in calculus prevalence. The differences between each group *by individual* were statistically significant (Chi-squared: for Joseon people and Russian settlers, *P* = 0.03859; for Joseon people and Siberian natives, *P* = 1.159e − 08; for Russian settlers and Siberian natives, *P* = 0.0002175) (Table 4). The differences between three groups *by teeth* were also statistically significant (Chi-squared: for Joseon people and Russian settlers, *P* = 9.669e − 10; for Joseon people and Siberian natives, *P* < 2.2e − 16; for Russian settlers and Siberian natives, *P* < 2.2e − 16). The Chi-squared test results for calculus prevalence by individual and teeth are summarized in Table 4. While there are many factors involved in the generation of calculus, diets have long been a part of major interest in related research [11, 13].

Joseon people and Russian settlers in this study must have had a nutrition based primarily on agricultural crops (mainly rice or wheat) with some meat, while Siberian natives mostly relied on animal products for their nutrition. The result of our study to compare agricultural (Joseon and Russian) and hunter-gatherer (native Siberians) populations suggests that an increase in carbohydrate intake might have had an important role in calculus formation in sixteenth to eighteenth century Eurasian continent.

**Figure 2:**
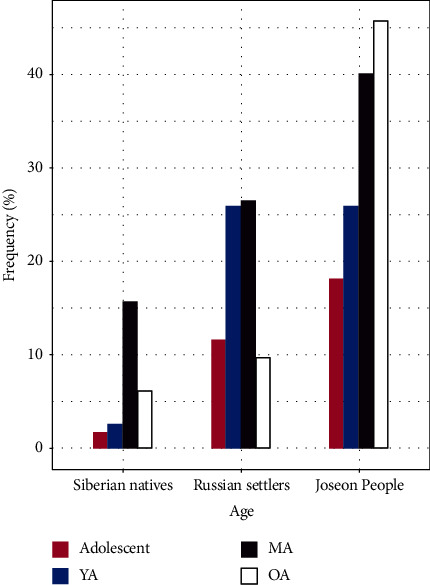
The analysis of calculus rates by age. Red, blue, black, and white bars indicate adolescents, young adults, middle adults, and old adults in each group, respectively. The calculus prevalence per teeth generally increases as age increases in Siberian natives, Russian setters, and Joseon people groups. This phenomenon is particularly remarkable in the process of aging from adolescence to middle adult, but is decreasing in old adult.

As for the analysis of calculus rates by age, as seen in Figure 2, the calculus prevalence *per teeth* generally increases as age increases in all Siberian native, Russian setters, and Joseon period groups. This phenomenon is particularly noticeable in the process of aging from adolescence to middle adult. However, we also note that the prevalence of calculus *per teeth*, which continued to increase until the middle adult, has been observed to decrease in old adult (Figure 2). We conjecture that this might have been caused by the effect of antemortem tooth loss in old age on dental calculus. This increasing pattern as age increases was not remarkable in the calculus prevalence *per individual*.

As for the analysis of calculus rates by sex, the prevalence of calculus deposition in males was noteworthy in all three groups (Table 5). In Siberian natives, calculus was found in 47 out of 422 male teeth (11.1%) while 7 out of 398 female teeth (1.8%). The calculus prevalence was statistically different between both sexes (Chi-squared test, *P* = 1.358e − 07). In case of Russian settler's teeth, we found 141 calculi out of 565 male teeth (25.0%) and 154 calculi out of 739 female teeth (20.8%). The difference was not statistically significant (Chi-squared test, *P* = 0.09028) (Table 5). In case of Joseon people, calculus was observed in 392 out of 1,112 male teeth (35.3%), while 256 out of 880 female teeth (29.1%). The difference in calculus frequency between sex was statistically confirmed (Chi-squared test, *P* = 0.00415) (Table 5). Higher prevalence of calculus in males than females appeared that the former had a dietary or behavioral practice that contributed to calculi formation or perhaps poorer oral hygiene than their female counterparts.

As mentioned above, in anthropological studies on past populations, it has been proposed that diets or subsistence strategy had impact on the prevalence of dental calculus [7, 10–20]. Such calculus-related studies have been also performed on modern people [2]. For instance, Gaare et al.'s report [52] on twentieth century student groups from Norway and Indonesia, for which variables like age, sex, and tooth brushing could be easily controlled, is significant to understand the impacts of diets or subsistence strategy on dental calculus rates. According to the study, Indonesian students showed significantly higher calculus rate than that of the Norwegian students, which might be caused by their characteristics in diets, drinking waters, or eating habits [52]. The authors also speculated that diets can be the most significant causing factor in the formation of dental calculus considering that the Norwegian individual with Asian cultural background (e.g., dietary habit) represented higher calculus rate than the other Norwegians [52]. These preceding studies on archaeological and modern populations are certainly helpful in interpreting results from the current report.

## 4. Conclusion

This study on the prevalence of dental calculus is conducted on three different populations in the sixteenth to nineteenth century Eurasian continent: Siberian natives, Russian settlers, and Joseon period Korean people. All these groups maintained different diet strategies as wheat- and rice-farming agriculturalists and hunter-gatherers. In brief, Joseon period Koreans were living in rice-cultivating society of East Asia. Russian settlers were based primarily on wheat as a major crop with meats. Siberian natives mostly relied on animal products as hunter-gatherers. The current study demonstrated a significant difference of calculus formation between those three groups with different diets or subsistence strategy in Eurasian continent. Higher prevalence of dental calculus was observed in agriculturalist Joseon Koreans and Russian settlers, but Siberian natives exhibited relatively lower frequency of dental calculus. The results of this study enable us to consider the meaning of association between diets, subsistence strategy, and dental calculus in different populations in history once again.

## Figures and Tables

**Table 1:** Archaeological information.

People	Site	Date	N	Activity and subsistence
Siberian natives				
Tatar	Omsk	17th to 20th century	34	Fishers-hunters, cattle breeder, farmers to a lesser extent
Khanty	Khanty-Mansi autonomous Okrug	17th to 18th century	7	Fishers-hunters
Nenet	Yamalo-Nenets autonomous Okrug	19th to 20th century	12	Fishers, reindeer herders
Russian settlers				
Russian	Omsk (Izyuk)	16th to 18th century	79	Agricultural (wheat)
Joseon period people				
Korean	South Korea	16th to 19th century	90	Agricultural (rice)

**Table 2:** Calculus prevalence of Siberian natives, Russian settlers, and Joseon period people (per individual).

Age	Siberian natives				Russian settlers				Joseon people			
	Total (*n*)	Affected (*n*)	Nonaffected (*n*)	Frequency (%)	Total (*n*)	Affected (*n*)	Nonaffected (*n*)	Frequency (%)	Total	Affected (*n*)	Nonaffected (*n*)	Frequency (%)
Adolescent	7	1	6	14.3	9	4	5	44.4	4	3	1	75.0
YA	26	6	20	23.1	30	23	7	76.7	38	27	11	71.1
MA	16	6	10	37.5	26	16	10	61.5	37	31	6	83.8
OA	4	1	3	25.0	14	5	9	35.7	11	8	3	72.7
Total	53	14	39	26.4	79	48	31	60.8	90	69	21	76.7

**Table 3:** Calculus prevalence of Siberian natives, Russian settlers, and Joseon period people (*per teeth*).

Age	Siberian natives				Russian settlers				Joseon people			
	Total (*n*)	Affected (*n*)	Nonaffected (*n*)	Frequency (%)	Total (*n*)	Affected (*n*)	Nonaffected (*n*)	Frequency (%)	Total	Affected (*n*)	Nonaffected (*n*)	Frequency (%)
Adolescent	121	2	119	1.7	138	16	122	11.6	116	21	95	18.1
YA	391	10	381	2.6	611	158	453	25.9	942	244	698	25.9
MA	242	38	204	15.7	400	106	294	26.5	783	314	469	40.1
OA	66	4	62	6.1	155	15	140	9.7	151	69	82	45.7
Total	820	54	766	6.6	1,304	295	1,009	29.2	1,992	648	1,344	32.5

**Table 4:** Statistical analysis of dental calculus prevalence between Siberian natives, Russian settlers, and Joseon people (Chi-squared test; *P* value).

	Siberian natives	Russian settlers	Joseon people
Siberian natives		^a^ < 2.2e-16∗∗∗∗	^a^ < 2.2e-16∗∗∗∗
Russian settlers	^a^ 0.0002175∗∗∗		^a^ 9.669e-10∗∗∗∗
Joseon people	^a^1.159e-08∗∗∗∗	^a^ 0.03859∗	

*Per individual* for lower left cells; *per teeth* for upper right cells. ^a^Chi-squared test.

**Table 5:** Prevalence of calculus per teeth by sex (Chi-squared test).

	Male			Female			*P* value
	Affected (*n*)	Non-affected (*n*)	Frequency (%)	Affected (*n*)	Nonaffected (*n*)	Frequency (%)	
Siberian natives	47	375	11.1	7	391	1.8	^a^1.358e-07∗∗∗∗
Russian settlers	141	424	25.0	154	585	20.8	^a^ 0.09028
Joseon people	392	720	35.3	256	624	29.1	^a^ 0.00415∗∗

^a^Chi-squared test.

## Data Availability

The datasets used in the current study are available from corresponding author on reasonable request.

## Supplementary Materials

Supplementary 1 Supplementary Table 1. Proportion of age in Siberian natives, Russian settlers, and Joseon people.

Supplementary 2 Supplementary Table 2. Proportion of sex in Siberian natives, Russian settlers, and Joseon people.
