# Supplementary material for: Dental Calculi of Siberian Natives, Russian Settlers, and Korean People of Joseon Dynasty Period in the 16th to 19th Century Eurasia Continent
Source: Biomed Res Int. 2022 May 9;2022:5765604. doi: 10.1155/2022/5765604 (PMC9112181; doi:10.1155/2022/5765604)
Supplement: Supplementary 1 — Supplementary Table 1. Proportion of age in Siberian natives, Russian settlers, and Joseon people. [file 5765604.f1.docx]

**Supplementary Table 1:** Proportion of Age in Siberian Natives, Russian settlers, and Joseon People

| Age | Groups | | |
| --- | --- | --- | --- |
|  | Siberian Natives | Russian Settlers | Joseon People |
| Adolescent | 7 | 9 | 4 |
| Young Adult | 26 | 30 | 38 |
| Middle Adult | 16 | 26 | 37 |
| Old Adult | 4 | 14 | 11 |
