# Supplementary material for: Dental Calculi of Siberian Natives, Russian Settlers, and Korean People of Joseon Dynasty Period in the 16th to 19th Century Eurasia Continent
Source: Biomed Res Int. 2022 May 9;2022:5765604. doi: 10.1155/2022/5765604 (PMC9112181; doi:10.1155/2022/5765604)
Supplement: Supplementary 2 — Supplementary Table 2. Proportion of sex in Siberian natives, Russian settlers, and Joseon people. [file 5765604.f2.docx]

**Supplementary Table 2:** Proportion of sex in Siberian Natives, Russian settlers, and Joseon People

| Groups | Female | Male |
| --- | --- | --- |
| Siberian Natives | 30 | 23 |
| Russian Settlers | 47 | 32 |
| Joseon People | 42 | 48 |
